# The Effect of Bicarbonate Administration via Continuous Venovenous Hemofiltration on Acid-Base Parameters in Ventilated Patients

**DOI:** 10.1155/2015/901590

**Published:** 2015-01-08

**Authors:** Andrew S. Allegretti, Jennifer E. Flythe, Vinod Benda, Emily S. Robinson, David M. Charytan

**Affiliations:** ^1^Division of Nephrology, Department of Medicine, Massachusetts General Hospital, 7 Whittier Place, Suite 106, Boston, MA 02114, USA; ^2^Division of Nephrology and Hypertension, Department of Medicine and Genetics, UNC School of Medicine, UNC Kidney Center, 7024 Burnett-Womack, CB No. 7155, Chapel Hill, NC 27599-7155, USA; ^3^Renal Division, Department of Medicine, Brigham and Women's Hospital, 75 Francis Street, MRB4, Boston, MA 02115, USA

## Abstract

*Background*. Acute kidney injury (AKI) and metabolic acidosis are common in the intensive care unit. The effect of bicarbonate administration on acid-base parameters is unclear in those receiving continuous venovenous hemofiltration (CVVH) and mechanical ventilatory support. *Methods*. Metabolic and ventilatory parameters were prospectively examined in 19 ventilated subjects for up to 96 hours following CVVH initiation for AKI at an academic tertiary care center. Mixed linear regression modeling was performed to measure changes in pH, partial pressure of carbon dioxide (*p*CO_2_), serum bicarbonate, and base excess over time. *Results*. During the 96-hour study period, *p*CO_2_ levels remained stable overall (initial *p*CO_2_ 42.0 ± 14.6 versus end-study *p*CO_2_ 43.8 ± 16.1 mmHg; *P* = 0.13 for interaction with time), for those with initial *p*CO_2_ ≤40 mmHg (31.3 ± 5.7 versus 35.0 ± 4.8; *P* = 0.06) and for those with initial *p*CO_2_ >40 mmHg (52.7 ± 12.8 versus 53.4 ± 19.2; *P* = 0.57). *p*CO_2_ decreased during the immediate hours following CVVH initiation (42.0 ± 14.6 versus 37.3 ± 12.6 mmHg), though this change was nonsignificant (*P* = 0.052). *Conclusions*. We did not detect a significant increase in *p*CO_2_ in response to the administration of bicarbonate via CVVH in a ventilated population. Additional studies of larger populations are needed to confirm this finding.

## 1. Background

Acute kidney injury (AKI) in the critically ill is common and associated with poor prognosis [1]. Metabolic acidosis occurs frequently among those with AKI and is independently associated with poor prognosis [2–4]. Metabolic acidosis is often treated with sodium bicarbonate to mitigate complications such as low vascular tone, depressed cardiac contractility, and increased incidence of arrhythmias [5]. Despite this common practice, there is minimal evidence showing a benefit to bicarbonate administration, and there are several studies that suggest harm. As a result, there is a lack of provider consensus on best practice [6–11].

The potential harm of bicarbonate administration has a plausible physiologic basis. First, delivery of bicarbonate comes at the expense of an added sodium load that can contribute to hypervolemia and further impair ventilation [12, 13]. In addition, bicarbonate may be dehydrated to peripheral carbon dioxide. Peripheral carbon dioxide requires adequate pulmonary function and gas exchange to be expired by the respiratory system [14]. The effects of bicarbonate depend, in part, on the nonbicarbonate buffering capacity of the cellular environment, namely, the partial pressure of carbon dioxide (*p*CO_2_) [15]. Several experimental and clinical studies have shown that in certain situations the administration of peripheral sodium bicarbonate can increase* p*CO_2_, thus underscoring the importance of adequate respiratory reserve in patients with acidemia [16–18]. However, none of these studies were conducted in the setting of renal failure requiring continuous renal replacement therapy.

Patients who require both renal replacement and ventilator support highlight the complexities inherent in acidemia and AKI. Renal replacement in the critically ill is often performed via continuous venovenous hemofiltration (CVVH). CVVH has three distinct physiologic effects on acid-base balance: (1) removal of unmeasured anions through convection, (2) addition of base (bicarbonate) by replacement infusion, and (3) ultrafiltration, which can impact gas exchange [19, 20]. As a result, studies have shown mixed effects of CVVH on acid-base parameters [21–31]. However, there is a paucity of evidence examining the effect of bicarbonate administration via CVVH in a ventilated population. We therefore undertook this study to analyze the impact of bicarbonate administration via CVVH on acid-base parameters and ventilation status over the initial 96 hours of CVVH. CVVH is widely used in the critically ill and in those with mixed respiratory and metabolic acidosis. If there was a large negative impact of bicarbonate administration in this setting, we might have expected a change in practice pattern in this cohort. We therefore hypothesized that bicarbonate administration via CVVH would not increase* p*CO_2_ levels.

## 2. Methods

### 2.1. Patient Population

We prospectively examined critically ill patients in the intensive care unit (ICU) of Brigham and Women's Hospital, a 793-bed, academic tertiary care center in Boston, Massachusetts, with eight specialized medical and surgical ICUs. From August 2011 to February 2012, potential subjects were identified by the nephrology ICU consult team at the time of initiation of CVVH. The treating nephrologist determined the need for CVVH and its prescription parameters including replacement fluid composition, rate, and goal ultrafiltration. Inclusion criteria included age >18 years, presence of acute kidney injury requiring CVVH therapy, and presence of mechanical ventilation at the start of enrollment. Exclusion criteria included history of any renal replacement therapy prior to ICU admission, presence of chronic tracheostomy, history of traumatic or anoxic brain injury, and history of end stage renal disease.

All data analyzed in this study were collected as part of the routine clinical care for each subject. Through review of medical records and CVVH and ICU flow sheets, a member of the research team collected data prospectively between time zero (start of CVVH) and hours 8, 16, 24, 36, 48, 60, 72, and 96 of receiving CVVH. Relevant data included demographic information, vital signs, ventilator settings, CVVH settings, comorbidities, active diagnoses, and laboratory data.

### 2.2. Description of CVVH

CVVH was performed via double-lumen catheters using machines from NxStage Medical (Lawrence, MA, USA) with prefilter fluid replacement. Target replacement fluid rates were typically 20 to 30 mL/kg/hour. A 1 : 1 nursing ratio was employed for all patients on CVVH. CVVH was performed according to the local standard of care with a preference for CVVH over intermittent dialysis for patients with hemodynamic instability or for those receiving vasopressor support. CVVH replacement fluids used during the study period are described in Table 1.

### 2.3. Analysis of Laboratory Tests

Acid-base parameters and partial pressures were calculated on arterial blood in heparinized syringes whereas electrolytes, including bicarbonate levels, were drawn from venous blood in tubes containing EDTA. All blood samples were measured in the clinical laboratory using standard clinical techniques.

### 2.4. Statistical Analysis

Baseline characteristics of subjects overall and those stratified by partial arterial pressure of carbon dioxide at time of continuous venovenous hemofiltration initiation were summarized as mean ± standard deviation (SD) or number (percentage). Acid-base parameters and electrolytes are shown as mean ± SD, median (25th, 75th percentile), and minimum-maximum values based on normality diagnostics and clinical appropriateness. Outcomes (change in serum pH,* p*CO_2_, HCO_3_, and base excess) used a maximum-likelihood, mixed-effects repeated-measures model (MMRM) with all longitudinal observations. Time was modeled as a continuous variable. Standard diagnostics for normality were performed on baseline data, outcomes, and outcome residuals, which supported normality assumptions and the validity of parametric regression analysis. Univariate regression models were constructed to evaluate the effect of potential confounders. As this was a pilot study, the sample size was too small for multivariable model adjustment. Sensitivity analyses were performed to evaluate the effect of removing one subject who received peripheral isotonic bicarbonate solution and one subject who received citrate based replacement fluid. STATA version 13 (College Station, Texas) was used for all statistical analysis. Two-tailed *P* values <0.05 were considered statistically significant.

### 2.5. Ethics Statement

The institutional review board for human subjects of the study hospital approved the study. All clinical investigation was conducted according to the principles expressed in the Declaration of Helsinki. All data collected for this study was done via review of medical record. No direct contact was made between members of the research team and any subject. The institutional review board waived the need for informed consent.

## 3. Results

Demographic, clinical, and biochemical characteristics of the study population are shown in Table 2. We studied 19 patients with a mean age of 57.5 years; 13 (68.4%) were male. The most common admitting diagnoses were respiratory failure/pneumonia (37%), heart failure/cardiogenic shock (32%), and sepsis (26%). At the time of CVVH initiation, 74% of subjects were vasopressor dependent, mean SOFA score was 13.4 ± 3.5, and mean lactate was 4.1 ± 4.9 mmol/L. One subject (5%) was extubated at hour 48. Two subjects (11%) died during the study period. All other subjects remained on CVVH until the end of the study period.

The mean pH at CVVH initiation was 7.32 ± 0.12. Nine subjects (47%) had an initial* p*CO_2_ ≤40 mmHg (mean initial pH 7.40 ± 0.06), nine subjects (47%) had an initial* p*CO_2_ >40 mmHg (mean initial pH 7.24 ± 0.12), and one subject did not have an initial blood gas available at the time of CVVH initiation. Mean blood urea nitrogen (BUN) decreased from 68.4 ± 35.0 mg/dL at CVVH initiation to 29.4 ± 14.2 at the end of the study period (*P* < 0.001). Mean creatinine decreased from 3.9 ± 1.2 mg/dL at CVVH initiation to 1.7 ± 0.7 mg/dL at the end of the study period (*P* < 0.001; Table 3).

Bicarbonate based replacement solution (32 mEq/L) was used in 18 of 19 subjects (95%). The remaining subject received citrate based replacement solution (citrate concentration 40 mEq/L). Across the entire study period, mean blood flow rate was 238 ± 33 mL/min, mean replacement solution flow rate was 2214 ± 484 mL/h, and mean ultrafiltration rate was 45 ± 62 mL/h (Table 4).

The majority of subjects were supported on assist control or pressure control ventilation at the time of enrollment (90%), but this decreased to 41% by hour 96. Across the 96-hour study duration, there were no significant changes in PEEP (*P* = 0.25), achieved tidal volume (*P* = 0.95), ventilator-set respiratory rate (*P* = 0.15), subject respiratory rate (*P* = 0.60), or FIO_2_ (*P* = 0.10; Table 5).

Values of acid-base parameters and electrolytes across each time point are detailed in Table 3. During the 96-hour study period,* p*CO_2_ levels remained stable overall (initial* p*CO_2_ 42.0 ± 14.6 versus end-study* p*CO_2_ 43.8 ± 16.1 mmHg; *P* = 0.13 for interaction with time). Neither the low initial* p*CO_2_ subgroup (≤40 mmHg) nor the high initial* p*CO_2_ subgroup (>40 mmHg) had a significant change in* p*CO_2_ during the 96-hour study period (31.3 ± 5.7 versus 35.0 ± 4.8, *P* = 0.06, and 52.7 ± 12.8 versus 53.4 ± 19.2, *P* = 0.57, resp.). There was a decrease in* p*CO_2_ during the immediate hours following CVVH initiation (42.0 ± 14.6 mmHg at hour 0 versus 37.3 ± 12.6 mmHg at hour 8), though this change was nonsignificant (*P* = 0.052). Results were qualitatively similar in separate secondary analyses excluding the subject who received citrate replacement solution and the subject who received three liters of additional isotonic sodium bicarbonate intravenous fluid.

**Figure 1:**
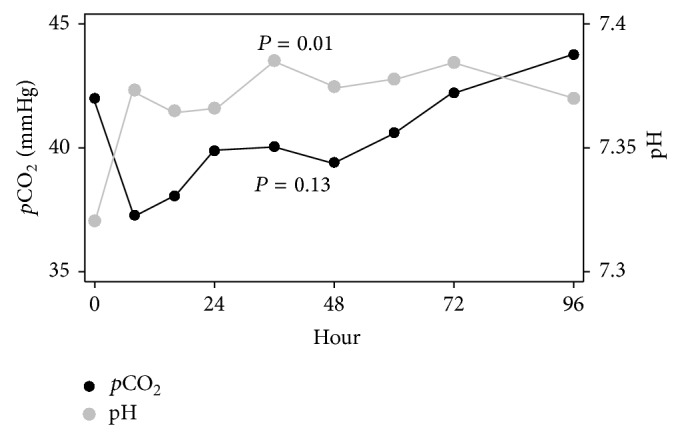
Changes in mean partial arterial pressure of carbon dioxide (*p*CO_2_) and pH over time.

Across all subjects, there was a nonsignificant (*P* = 0.12) increase in pH for the 96-hour study duration (Figure 1). pH increased from 7.32 ± 0.12 at hour 0 to 7.37 ± 0.10 at hour 8 (*P* = 0.01). For serum HCO_3_ levels, there was a significant (*P* = 0.003) increase across the 96-hour study period (see Supplementary Figure in the Supplementary Material available online at http://dx.doi.org/10.1155/2015/901590). There was no significant change in HCO_3_ between hour 0 and hour 8 (*P* = 0.57). For base excess, there was a significant (*P* = 0.005) increase across the 96-hour study period. There was no significant change in base excess between hour 0 and hour 8 (*P* = 0.13).

## 4. Discussion

We studied the effects of large volume bicarbonate infusion via CVVH in a ventilated cohort and found that there was no significant change in* p*CO_2_ in response to bicarbonate administration across a 96-hour period of observation. During the first 8 hours of CVVH use, there was a significant improvement in acidemia and a trend toward a decrease in* p*CO_2_. This nonsignificant decrease in* p*CO_2_ disappeared over the course of 96 hours. While this was observed in a small sample, it provides preliminary data for hypothesis generation and supports further study of a clinically relevant impact in acid-base handling in the ICU.

The impact of CVVH on acid-base parameters is important. Like in prior work, our study relies on* p*CO_2_ as a clinical marker of total tissue carbon dioxide content, which is the ultimate driver of hypercapnia. While the delivery of a buffer (usually bicarbonate) during CVVH is relied upon to treat metabolic acidosis [19], clinicians are often concerned about a potential compensatory increase in* p*CO_2_, especially in patients with limited respiratory reserve. However, prior analyses examining the effect of renal replacement on acid-base parameters have had inconsistent results across varied clinical scenarios. Rocktäschel et al. published a study of 40 subjects receiving CVVH and reported increases in pH,* p*CO_2_, and bicarbonate levels over 3 daily lab measurements [21]. Compared to this study, our study provides more granularity with additional time points and an extended follow-up period. Our results do not confirm their findings. This may be due to differences in sample characteristics (the ventilation status of their cohort was not reported) and delivery of CVVH (the buffer used for CVVH in their study was lactate, rather than bicarbonate) [21]. Tan et al. compared lactate and bicarbonate replacement solution for CVVH in eight patients over 120 minutes and did not show differences in any acid-base parameters [30]. This may not have been a long enough observation period to detect significant changes. Neither of these two papers reflects current clinical practice, as the use of lactate as a buffer in CVVH replacement fluid has been largely replaced by bicarbonate. Symreng et al. examined seven nonventilated subjects on high efficiency dialysis over 6 hours and showed an increase in tidal volume but not* p*CO_2_. These findings lead to the conclusion that a healthy ventilatory response is required to excrete the* p*CO_2_ generated by infusion of a buffer [29]. It is hard to apply these results to critically ill patients given this was not a study performed in the ICU setting.

Our study is the first to examine the acid-base parameters in a cohort simultaneously receiving CVVH and uniformly receiving mechanical ventilatory support. This sample was selected to examine the effects of high-rate delivery of bicarbonate or bicarbonate equivalents (citrate) in patients with impaired respiratory reserve. This cohort was at increased risk for hypercarbia due to the conversion of bicarbonate into carbon dioxide. Given the lack of significant increase in* p*CO_2_ across the initial 96 hours of CVVH, this suggests that the effect of bicarbonate on* p*CO_2_ may not be substantial even in a critically ill, ventilated population. Although our sample size was small, a subgroup analysis of individuals with an initial* p*CO_2_ above 40 mmHg was reassuring. No significant change in* p*CO_2_ was seen in this group with the most impaired gas exchange. There were only 3 patients with initial* p*CO_2_ ≥65 mmHg, so it is not clear if these results would apply to those with extreme hypercarbia. Furthermore, there were no patients in this sample undergoing simultaneous extracorporeal carbon dioxide removal (e.g., ECMO devices). Those with such extreme physiology warrant further study, as the use of both circuits in series for patients who have concurrent severe metabolic and respiratory acidosis is not clearly defined. Nevertheless, this further suggests that the physiologic effects of bicarbonate are tolerable even in those with concurrent respiratory acidosis.

It was surprising that during the first eight-hour interval there was an initial drop in* p*CO_2_ in response to starting CVVH that approached statistical significance. This is opposite of the expected physiologic impact of bicarbonate administration on* p*CO_2_ [16–18]. Several factors could account for this initial decrease, including transcellular shifts of* p*CO_2_, concurrent ultrafiltration (which could result in improved gas exchange given a mean 45 mL/hour ultrafiltration rate for all subjects), or other unmeasured properties of this population. Nevertheless, the observation that the* p*CO_2_ levels decreased initially is reassuring and suggests that even in this population there is likely to be sufficient native or assisted ventilatory reserve to compensate appropriately for changes in* p*CO_2_ generation following bicarbonate administration.

This sample did not include patients who received peripheral sodium bicarbonate without CVVH support. These results should not be extrapolated to patients not receiving renal replacement. While the same principles might apply to bicarbonate/carbon dioxide handling in all patients in the ICU regardless of renal replacement status, there are additional physiologic effects of CVVH, such as fluid removal via ultrafiltration and clearance of other toxins and inflammatory cytokines that may influence acid-base management. In addition, the gradual infusion of bicarbonate via CVVH (a mean change of only 3.4 mEq/dL serum HCO_3_ over the 96-hour study period) may permit time for respiratory compensation, which may be better tolerated than bolus delivery of ampules of sodium bicarbonate. However, this is speculative, and further study of patients not requiring renal replacement therapy is needed.

Our findings should be understood within the context of several limitations of the study design. The sample size was small, which limited the ability to control for multiple confounders in regression analysis and also limited power to detect small changes in* p*CO_2_. Confirmation in a larger cohort is needed, but* p*CO_2_ actually decreased during the first 8 hours, suggesting that the small size is of the cohort is an unlikely explanation for the failure to demonstrate an increasing trend in* p*CO_2_ during bicarbonate CVVH. Furthermore, meticulous measurements across standardized time intervals allowed us to characterize acid-base parameters in greater detail than previous analyses. Additionally, outcomes and observations were all physiologic measurements. This study was not designed or powered to look at the impact on clinical outcomes such as time on the ventilator, time on CVVH, or mortality, although those outcomes are clearly of great interest. We studied a cohort with a greater severity of illness than the average ICU patient [32]. Thus, our results may not be generalizable to less severely ill patients, although the group we analyzed would be more likely to exhibit consequences of bicarbonate-induced hypercarbia than less severely ill patients. Additionally, there were not enough subjects with* p*CO_2_ >65 mmHg to draw definitive conclusions about those with severe hypercarbia. Finally, the amount of CVVH downtime was not recorded as part of the routine clinical care of these subjects. This factor could have altered bicarbonate delivery. However, the significant decrease observed in BUN and Cr suggests that downtime was minimal over the 96-hour observation period.

We conclude that, in this sample, there was no significant increase in* p*CO_2_ in response to the administration of large volumes of bicarbonate via CVVH in this ventilated population. This finding should be confirmed in a larger study that can be powered to adjust for multiple potential confounders of the association. Our results suggest that a clinically relevant increase in* p*CO_2_ is not an inevitable complication of the administration of bicarbonate through CVVH in ventilated patients. There remains a need for future studies to characterize the complex acid-base interactions in patients receiving CVVH, peripheral bicarbonate, and ventilator support.

## Supplementary Material

Changes in mean partial pressure of carbon dioxide (*p*CO_2_) and serum bicarbonate (HCO_3_) over time. There was no significant change *p*CO_2_ over the 96-hour study period. There was an increase in HCO_3_ over the 96-hour study period.

## Figures and Tables

**Table 1:** Continuous venovenous hemofiltration replacement solution composition.

	Bicarbonate	Bicarbonate	Bicarbonate	Citrate
	4K^+^/2.5Ca^++^	2K^+^/0Ca^++^	0K^+^/2.5Ca^++^	
Sodium (mmol/L)	140	140	140	145
Potassium (mmol/L)	4.0	2.0	0	0.0
Magnesium (mEq/L)	1.5	1.0	1.5	1.5
Calcium (mmol/L)	2.5	0	2.5	0
Chloride (mmol/L)	113	111.5	109	105
Dextrose (mg/dL)	100	100	100	200
Citrate (mmol/L)	0	0	0	40
Bicarbonate (mmol/L)	32	32	32	0
Lactate (mmol/L)	3.0	3.0	3.0	0

**Table 2:** Baseline patient characteristics by partial arterial pressure of carbon dioxide at time of continuous venovenous hemofiltration initiation^a^.

	Patients	*p*CO_2_ ≤ 40 mmHg	*p*CO_2_ > 40 mmHg
	(*n* = 19)^b^	(*n* = 9)	(*n* = 9)
Age	57.5 ± 15.4	61.8 ± 17.3	55.2 ± 13.0
Male	13 (68.4)	6 (66.7)	7 (77.8)
ICU type			
Medical	7 (36.8)	3 (33.3)	3 (33.3)
Surgical^c^	7 (36.8)	4 (44.5)	3 (33.3)
Cardiac	5 (26.2)	2 (22.2)	3 (33.3)
ICU admission diagnosis			
Sepsis	5 (26.3)	2 (22.2)	2 (22.2)
Heart failure/cardiogenic shock	6 (31.6)	1 (11.1)	5 (55.6)
Respiratory failure/pneumonia	7 (36.8)	5 (55.6)	2 (22.2)
Other causes^d^	1 (5.3)	1 (11.1)	0
Preexisting comorbid condition			
Hypertension	10 (52.6)	5 (55.6)	5 (55.6)
Coronary artery disease	5 (26.3)	1 (11.1)	4 (44.4)
Chronic kidney disease^e^	7 (36.8)	2 (22.2)	5 (55.6)
Diabetes mellitus	10 (52.6)	2 (22.2)	7 (77.8)
Liver disease	1 (5.3)	0	0
COPD	6 (31.5)	3 (33.3)	2 (22.2)
Cancer	7 (36.8)	3 (33.3)	4 (44.4)
Systolic blood pressure (mmHg)	95.6 ± 16.2	100.8 ± 14.4	89.3 ± 17.3
Vasopressor dependency	14 (73.7)	6 (66.7)	8 (88.9)
Urine output (mL)^f^	14.6 (17.1)	12.6 ± 17.4	17.2 ± 18.4
SOFA score^g^	13.4 ± 3.5	12.8 ± 4.1	14.0 ± 2.7
pH^g^	7.32 ± 0.12	7.40 ± 0.06	7.24 ± 0.12
Creatinine (mg/dL)	3.9 ± 1.2	3.8 ± 1.0	3.8 ± 1.3
Blood urea nitrogen (mg/dL)	68.4 ± 35.8	57.2 ± 29.4	72.1 ± 36.3
Potassium (mmol/L)	4.9 ± 1.0	4.5 ± 1.2	5.2 ± 0.8
Lactate (mmol/L)^h^	4.1 ± 4.9	4.7 ± 5.6	3.4 ± 4.2
White blood cell count (K/*μ*L)	15.7 ± 6.6	15.6 ± 5.5	17.1 ± 7.1
Platelets (K/*μ*L)	135 ± 111	120 ± 90	163 ± 130
Mortality within 7 days of CVVH initiation	2 (10.5)	0	2 (22.2)

^a^Values presented as mean ± standard deviation or *n* (%).

^b^1 of 19 patients did not have a starting arterial blood gas.

^c^Including surgical, cardiothoracic, and cardiac surgery ICUs.

^d^Retroperitoneal bleed.

^e^Including 1 end stage renal disease patient on dialysis.

^f^Urine output in 1-hour period prior to starting CVVH.

^g^ *N* = 18.

^h^ *N* = 15 for all patients, *N* = 8 for *p*CO_2_ ≤ 40 mmHg, and *N* = 7 for *p*CO_2_ > 40 mmHg.

ICU: intensive care unit; CVVH: continuous venovenous hemofiltration; COPD: chronic obstructive pulmonary disease, K: 1000, and *p*CO_2_: partial arterial pressure of carbon dioxide.

**Table 3:** Acid-base parameters and electrolytes^a^.

	Hour 0	Hour 8	Hour 16	Hour 24	Hour 36	Hour 48	Hour 60	Hour 72	Hour 96
pH	7.32 ± 0.12	7.37 ± 0.08	7.37 ± 0.10	7.37 ± 0.08	7.39 ± 0.09	7.37 ± 0.08	7.38 ± 0.07	7.38 ± 0.07	7.37 ± 0.11
	(*n* = 18)	(*n* = 18)	(*n* = 18)	(*n* = 18)	(*n* = 19)	(*n* = 17)	(*n* = 18)	(*n* = 18)	(*n* = 17)

*p*CO_2_ (mmHg)	42.0 ± 14.6	37.3 ± 12.6	38.1 ± 9.7	39.9 ± 10.3	40.1 ± 11.6	39.4 ± 11.6	40.6 ± 10.3	42.2 ± 10.2	43.8 ± 16.1
	(*n* = 18)	(*n* = 18)	(*n* = 18)	(*n* = 18)	(*n* = 19)	(*n* = 17)	(*n* = 18)	(*n* = 18)	(*n* = 17)

HCO_3_ (mmol/L)^b^	19.8 ± 5.1	20.1 ± 5.1	19.7 ± 4.2	22.3 ± 3.9	22.8 ± 3.7	23.1 ± 3.2	22.8 ± 3.5	23.3 ± 2.4	23.2 ± 3.8
	(*n* = 19)	(*n* = 18)	(*n* = 18)	(*n* = 18)	(*n* = 19)	(*n* = 17)	(*n* = 18)	(*n* = 16)	(*n* = 16)

Base excess (mmol/L)	−5.2 ± 6.5	−4.2 ± 5.4	−3.9 ± 5.4	−3.3 ± 3.6	−2.2 ± 3.0	−2.3 ± 2.7	−1.8 ± 3.3	−1.2 ± 2.6	−1.4 ± 3.3
	(*n* = 18)	(*n* = 18)	(*n* = 18)	(*n* = 18)	(*n* = 19)	(*n* = 17)	(*n* = 18)	(*n* = 18)	(*n* = 17)

Sodium (mmol/L)	132.7 ± 6.5	132.8 ± 5.1	132.5 ± 5.6	132.5 ± 5.9	132.1 ± 3.9	132.1 ± 3.9	132.1 ± 4.2	132.4 ± 4.1	131.1 ± 3.8
	(*n* = 19)	(*n* = 18)	(*n* = 18)	(*n* = 18)	(*n* = 19)	(*n* = 17)	(*n* = 18)	(*n* = 16)	(*n* = 16)

Potassium (mmol/L)	4.9 ± 1.0	4.6 ± 0.8	4.6 ± 0.7	4.6 ± 0.7	4.5 ± 0.6	4.5 ± 0.8	4.4 ± 0.4	4.3 ± 0.5	4.5 ± 0.5
	(*n* = 19)	(*n* = 19)	(*n* = 18)	(*n* = 19)	(*n* = 19)	(*n* = 19)	(*n* = 18)	(*n* = 17)	(*n* = 16)

Chloride (mmol/L)	96.4 ± 6.2	97.2 ± 5.7	97.4 ± 4.5	97.3 ± 5.4	98.5 ± 4.6	99.2 ± 3.5	98.2 ± 4.1	99.3 ± 3.7	98.8 ± 4.0
	(*n* = 19)	(*n* = 18)	(*n* = 18)	(*n* = 18)	(*n* = 19)	(*n* = 17)	(*n* = 18)	(*n* = 16)	(*n* = 16)

Ionized calcium (mg/dL)	1.03 ± 0.1	1.08 ± 0.3	1.05 ± 0.1	1.04 ± 0.1	1.03 ± 0.1	1.08 ± 0.1	1.09 ± 0.1	1.11 ± 0.1	1.11 ± 0.1
	(*n* = 19)	(*n* = 16)	(*n* = 17)	(*n* = 19)	(*n* = 18)	(*n* = 16)	(*n* = 18)	(*n* = 16)	(*n* = 15)

BUN (mg/dL)	68.4 ± 35.8	60.2 ± 33.2	51.8 ± 27.1	45.4 ± 22.5	40.1 ± 20.2	37.5 ± 18.9	34.1 ± 16.4	30.5 ± 15.2	29.4 ± 14.6
	(*n* = 19)	(*n* = 18)	(*n* = 18)	(*n* = 18)	(*n* = 19)	(*n* = 17)	(*n* = 18)	(*n* = 16)	(*n* = 16)

Creatinine (mg/dL)	3.9 ± 1.2	3.5 ± 1.2	3.1 ± 1.1	2.8 ± 1.0	2.4 ± 1.0	2.3 ± 0.8	2.0 ± 0.7	2.0 ± 0.7	1.7 ± 0.7
	(*n* = 19)	(*n* = 18)	(*n* = 18)	(*n* = 18)	(*n* = 19)	(*n* = 17)	(*n* = 18)	(*n* = 16)	(*n* = 16)

^a^Values presented as mean ± standard deviation.

^b^Drawn from venous chemistry panel.

*p*CO_2_: partial arterial pressure of carbon dioxide, HCO_3_: bicarbonate, and BUN: blood urea nitrogen.

**Table 4:** Continuous venovenous hemofiltration prescription characteristics.

	All subjects (*n* = 19)	Bicarbonate 4K^+^/2.5Ca^++^ (*n* = 10)	Bicarbonate 2K^+^/0Ca^++^ (*n* = 5)	Bicarbonate 0K^+^/2.5Ca^++^ (*n* = 3)	Citrate (*n* = 1)
Blood flow rate (mL/min)					
Mean ± SD	238 ± 33	238 ± 34	244 ± 17	247 ± 11	175 ± 46
Median [25th, 75th]	250 [250, 250]	250 [250, 250]	250 [250, 250]	250 [250, 250]	150 [150, 200]
(min, max)	(150, 250)	(150, 250)	(200, 250)	(200, 250)	(150, 250)
Solution flow rate (mL/h)					
Mean ± SD	2214 ± 484	2158 ± 462	2220 ± 527	2563 ± 453	2050 ± 256
Median [25th, 75th]	2000 [2000, 2400]	2000 [2000, 2400]	2000 [2000, 2400]	2500 [2000, 3000]	2000 [2000, 2200]
(min, max)	(2000, 3200)	(2000, 3200)	(2000, 3000)	(2000, 3000)	(1600, 2400)
Ultrafiltration rate (mL/h)					
Mean ± SD	45 ± 62	47 ± 63	39 ± 51	45 ± 53	50 ± 53
Median [25th, 75th]	0 [0, 100]	10 [0, 100]	0 [0, 100]	0 [0, 50]	50 [0, 100]
(min, max)	(0, 250)	(0, 250)	(0, 150)	(0, 250)	(0, 100)

Rates reflect average values over the course of the 96-hour study duration. *N* represents the number of subjects on a given solution prescription at the time of CVVH initiation.

**Table 5:** Ventilator settings.

	Hour 0	Hour 8	Hour 16	Hour 24	Hour 36	Hour 48	Hour 60	Hour 72	Hour 96
	(*N* = 19)	(*N* = 19)	(*N* = 19)	(*N* = 19)	(*N* = 19)	(*N* = 17)	(*N* = 17)	(*N* = 17)	(*N* = 16)
Mode									
AC or PC	17 (89.5%)	16 (84.2)	15 (78.9)	15 (78.9)	14 (73.7)	12 (70.6)	12 (70.6)	10 (58.8)	7 (41.2)
PS	2 (10.5%)	2 (10.5)	3 (15.8)	3 (15.8)	4 (21.0)	4 (21.0)	4 (21.0)	6 (35.3)	8 (47.1)
Oscillator^a^	0	1 (5.3)	1 (5.3)	1 (5.3)	1 (5.3)	1 (5.3)	1 (5.3)	1 (5.9)	1 (5.9)
PEEP (cm H_2_O)	8.0 ± 3.5	9.0 ± 6.0	9.0 ± 6.0	9.3 ± 6.0	10.0 ± 7.4	10.2 ± 7.7	10.7 ± 7.9	10.1 ± 7.7	9.1 ± 7.3
Achieved tidal volume (mL)	520 ± 128	519 ± 122	518 ± 130	520 ± 133	519 ± 122	506.1 ± 134.1	503.5 ± 156.0	495.4 ± 142.4	540.3 ± 162.5
		(*n* = 18)	(*n* = 18)	(*n* = 18)	(*n* = 18)	(*n* = 16)	(*n* = 16)	(*n* = 16)	(*n* = 15)
Ventilator RR (/minute)	22.0 ± 7.5	23.0 ± 8.3	23.0 ± 8.8	21.9 ± 8.2	22.2 ± 7.9	22.9 ± 7.6	22.3 ± 8.0	22.5 ± 8.1	20.7 ± 8.9
	(*n* = 18)								
Subject RR (/minute)	23.5 ± 8.8	23.5 ± 8.8	23.5 ± 8.6	22.7 ± 8.4	23.5 ± 7.6	24.8 ± 8.3	23.8 ± 9.1	24.3 ± 7.9	22.8 ± 9.6
FIO_2_ (%)	62.4 ± 24.5	58.7 ± 20.5	53.9 ± 20.0	53.4 ± 21.7	55.5 ± 23.1	54.2 ± 24.6	56.5 ± 24.5	53.8 ± 22.1	53.8 ± 22.1

^a^High frequency oscillator ventilator.

AC: assist control; PC: pressure control; PS: pressure support; PEEP: positive end expiratory pressure; RR: respiratory rate; FIO_2_: fraction of inspired oxygen.
