# Supplementary material for: The Effect of Bicarbonate Administration via Continuous Venovenous Hemofiltration on Acid-Base Parameters in Ventilated Patients
Source: Biomed Res Int. 2015 Jan 8;2015:901590. doi: 10.1155/2015/901590 (PMC4306401; doi:10.1155/2015/901590)

Supplementary Figure. Changes in mean partial arterial pressure of carbon dioxide (pCO_2) and serum bicarbonate (HCO_3) over time

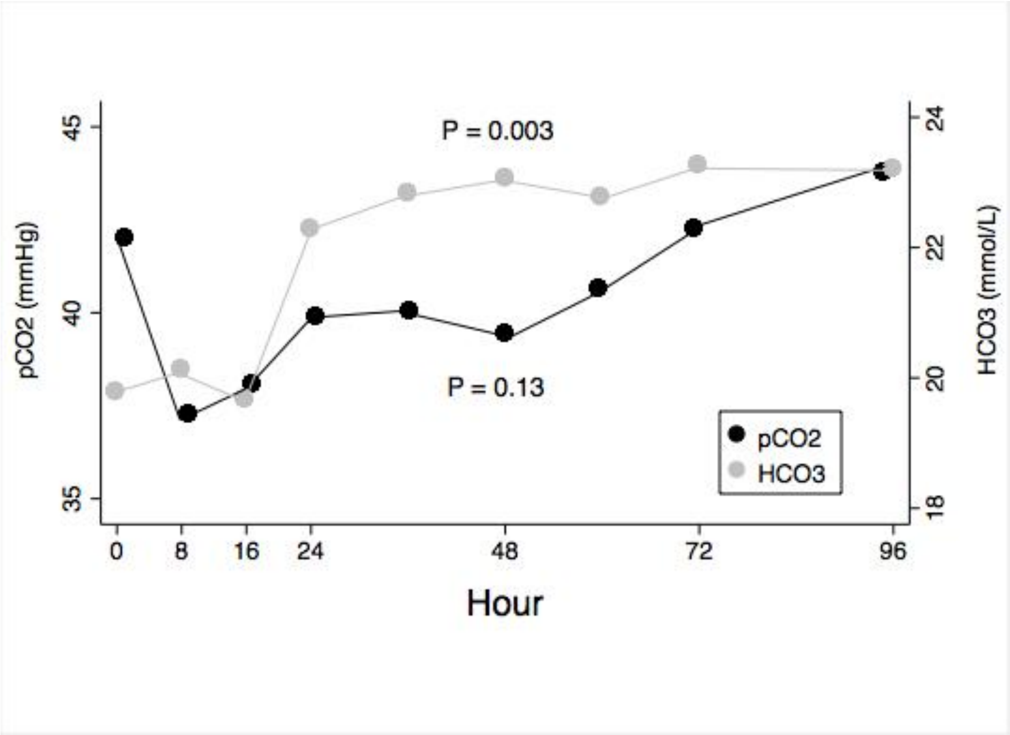

Supplement: Supplementary file 1 — Changes in mean partial pressure of carbon dioxide (pCO2) and serum bicarbonate (HCO3) over time. There was no significant change pCO2 over the 96-hour study period. There was an increase in HCO3 over the 96-hour study period. [file 901590.f1.pdf]
